# Reduction in skin cancer diagnoses in the UK during the COVID‐19 pandemic

**DOI:** 10.1111/ced.14411

**Published:** 2020-10-08

**Authors:** T. W. Andrew, M. Alrawi, P. Lovat

**Affiliations:** ^1^ Department of Plastic and Reconstructive Surgery Newcastle University Trust Hospitals Newcastle upon Tyne UK; ^2^ Translation and Clinical Research Institute Newcastle University Centre for Cancer Newcastle University Newcastle upon Tyne UK

## Abstract

The UK healthcare system, including skin cancer departments, has been profoundly affected by the COVID‐19 pandemic. Despite service capacity and a worldwide increase in incidence, anecdotal reports suggest a decline in skin cancer diagnoses following COVID‐19. To determine if there has been a decrease in skin cancer diagnosis in the UK in the COVID‐19 era, we analysed data from the Northern Cancer Network from 23 March 2020 to 23 June 2020 and compared it with the same period in 2019 (pre‐COVID). In the COVID period, there was a decrease of 68.61% in skin cancer diagnoses, from 3619 to 1136 (*P* < 0.01). Surprisingly, skin cancer waiting times were also reduced in the COVID period compared to the pre‐COVID period (median of 8 and 12 days, respectively; *P* < 0.001). Collectively, these data highlight a statistically significant reduction in both skin cancer diagnoses and waiting times during the COVID period.

The UK healthcare system has been profoundly affected by the COVID‐19 pandemic, which is currently responsible for over 45 000 deaths.^1^ Skin cancers are the most common cancer in white populations, and present significant morbidity and mortality. The British Association of Dermatologists (BAD) suggest using teledermatology to triage wait referrals for patients with suspected skin cancer and to book patients for surgery where possible during the current pandemic.^2^ However, despite service capacity and a worldwide increase in incidence, anecdotal reports suggest a decline in skin cancer diagnoses. We performed a study to assess changes in skin cancer diagnoses in the UK.

## Report

To determine if there has been a decrease in skin cancer diagnosis in the UK in the COVID‐19 era, we analysed data from the Northern Cancer Network from 23 March 2020 to 23 June 2020. The date of 23 March 2020 was identified as the beginning of the COVID‐19 period, corresponding with the start of the UK national lockdown and significantly affecting medical operations. We compared waiting times to the same period in 2019 (the pre‐COVID period) and calculated the percentage change in skin cancer diagnoses in the COVID and pre‐COVID periods.

**Figure 1:**
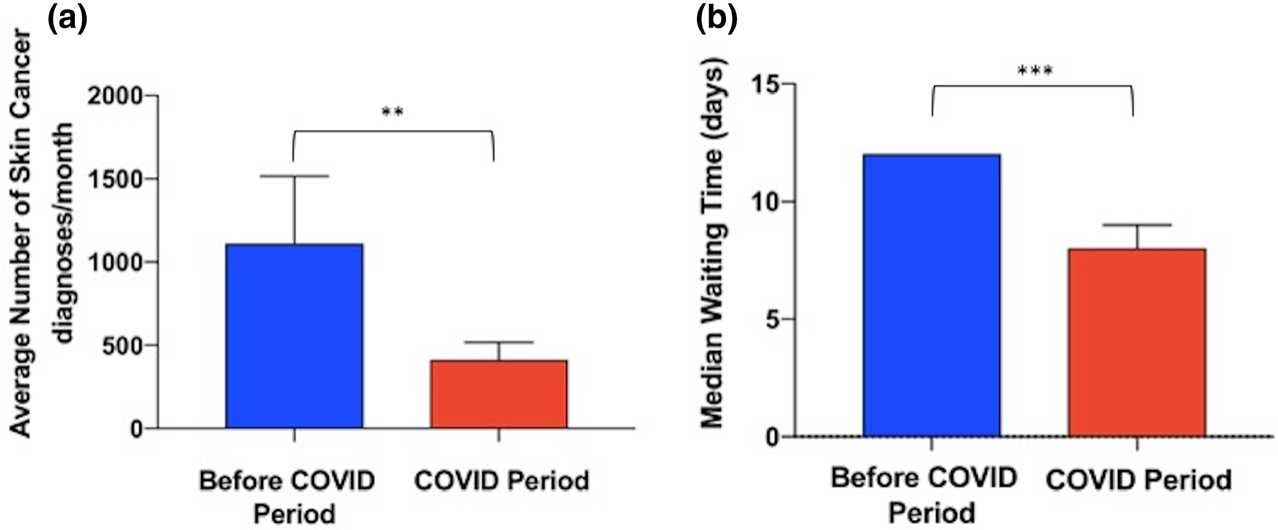
Comparison of skin cancer diagnoses and waiting times between the pre‐COVID and COVID periods. (a, b) Column charts representing (a) mean (and SEM) number of skin cancer diagnoses per month in a cohort of 4755 patients included in the Northern Cancer Network, and (b) median (and 95% CI) waiting time of skin cancer referrals to first appointment. **P < 0.01; ***P < 0.001.

In total, 3619 skin cancer diagnoses were made in the pre‐COVID period; however, in the corresponding COVID period there was a decrease of 68.61% in skin cancer diagnoses, down to 1136 (*P* < 0.01). Surprisingly, skin cancer waiting times were also reduced in the COVID compared with the pre‐COVID period (median 8 and 12 days respectively; *P* < 0.001). These findings are summarized in Fig. 1. Collectively these data highlight a statistically significant reduction in both skin cancer diagnoses and waiting times during the COVID period.

The COVID‐19 pandemic has affected global health trends.^3^ Our preliminary analysis of the first 3 months of the COVID pandemic shows a decrease in overall skin cancer diagnoses, and interestingly, a reduction in waiting time from referral to initial appointment in the UK. These findings suggest continued capacity in skin cancer services despite COVID‐19. Potential reasons for the reduction in waiting time include prioritization of cancer referrals and an overall decrease in skin cancer referrals from primary care. Additionally, the successful adoption of telemedicine has allowed appropriate triage of those lesions requiring face‐to‐face assessment by a specialist.^4^ The decrease in referrals may be due to patient reluctance to seek medical attention during the pandemic and may be confounded by challenges in primary care services. As the pandemic endures, it is particularly crucial for both doctors and patients to be aware of the decreasing presentation of skin cancers and shortened waiting times, in order to facilitate thoughtful and transparent decision‐making.^5^, ^6^

**Learning points**

- The BAD suggest using teledermatology to triage wait referrals for patients with suspected skin cancer and book patients for surgery where possible during the current COVID pandemic.
- There was a decrease of 68.61% in skin cancer diagnoses, from 3619 in the pre‐COVID period to 1136 in the COVID period (*P* < 0.01).
- Skin cancer waiting times were also reduced in the COVID compared with the pre‐COVID period (median 8 and 12 days respectively; *P* < 0.001).
- These findings suggest continued capacity in skin cancer services despite COVID‐19. This is potentially due to prioritization of cancer referrals and an overall decrease in skin cancer referrals from primary care.
- Additionally, successful adoption of telemedicine allowed appropriate triage of those lesions requiring face‐to‐face assessment by a specialist.
- As the pandemic endures, it is particularly crucial for both doctors and patients to be aware of the decreasing presentation of skin cancers and the shortened waiting times to facilitate thoughtful and transparent decision‐making.
